# The Role of Mechanically-Activated Ion Channels Piezo1, Piezo2, and TRPV4 in Chondrocyte Mechanotransduction and Mechano-Therapeutics for Osteoarthritis

**DOI:** 10.3389/fcell.2022.885224

**Published:** 2022-05-04

**Authors:** Winni Gao, Hamza Hasan, Devon E. Anderson, Whasil Lee

**Affiliations:** ^1^ Department of Pharmacology and Physiology, University of Rochester Medical Center, Rochester, NY, United States; ^2^ Department of Biomedical Engineering, University of Rochester, Rochester, NY, United States; ^3^ Department of Orthopaedics and Rehabilitation, University of Rochester Medical Center, Rochester, NY, United States; ^4^ Center for Musculoskeletal Research, University of Rochester Medical Center, Rochester, NY, United States

**Keywords:** chondrocyte, mechanotransduction, osteoarthritis, mechanically-activated calcium channels, Piezo1, Piezo2, TRPV4, mechano-therapeutics

## Abstract

Mechanical factors play critical roles in the pathogenesis of joint disorders like osteoarthritis (OA), a prevalent progressive degenerative joint disease that causes debilitating pain. Chondrocytes in the cartilage are responsible for extracellular matrix (ECM) turnover, and mechanical stimuli heavily influence cartilage maintenance, degeneration, and regeneration via mechanotransduction of chondrocytes. Thus, understanding the disease-associated mechanotransduction mechanisms can shed light on developing effective therapeutic strategies for OA through targeting mechanotransducers to halt progressive cartilage degeneration. Mechanosensitive Ca^2+^-permeating channels are robustly expressed in primary articular chondrocytes and trigger force-dependent cartilage remodeling and injury responses. This review discusses the current understanding of the roles of Piezo1, Piezo2, and TRPV4 mechanosensitive ion channels in cartilage health and disease with a highlight on the potential mechanotheraputic strategies to target these channels and prevent cartilage degeneration associated with OA.

## Introduction

Articular cartilage is a tissue that provides a low-friction surface for smooth movement of diarthrodial joints under mechanical loading. More than 300 million people globally and 35 million Americans are affected by osteoarthritis (OA), a debilitating disease with risk factors of increasing age, female sex, obesity, joint injuries, and overuse of joints (Buckwalter and Martin, 2006; Michael et al., 2010; Kloppenburg and Berenbaum, 2020; Yunus et al., 2020). The hallmark of OA is progressive cartilage degeneration, and patients with OA usually experience pain with everyday movement that can ultimately lead to a loss in function of the joint (Lawrence et al., 2008). OA patients also have an increased rate of comorbidities including obesity, diabetes, and cardiovascular disease, likely due to decreased physical activity resulting from loss in joint function (Suri et al., 2012; Muckelt et al., 2020). Numerous disease-modifying OA drugs (DMOADs) have been developed to reduce cartilage degeneration and joint discomfort, yet none have demonstrated long-term efficacy and safety (Hodgkinson et al., 2021; Oo et al., 2021).

Mechanical cues influence chondrocyte biosynthesis via mechanotransduction, a conversion process of mechanical stimuli into intracellular biochemical responses (Lee et al., 2000; Leong et al., 2011; Zhao et al., 2020). Chondrocytes are intrinsically mechanosensitive and sense a wide-range of mechanical loading due to the abundantly expressed mechanically-activated (MA) ion channels, including Piezo1, Piezo2, and TRPV4 (Suzuki et al., 2020; Xu et al., 2020; Delco and Bonassar, 2021). These channels are apart of the chondrocyte channelome, where different ion channels can play a role in regulating membrane potential, cell volume, intracellular pH, or mechanotransduction (Erickson et al., 2001; Barrett-Jolley et al., 2010; Mobasheri et al., 2019). A large gradient in Ca^2+^ concentration is maintained at rest, where Ca^2+^ is more abundant extracellularly than intracellularly, allowing Ca^2+^ influx into chondrocytes upon activation of MA channels (Lv et al., 2018). Mechanical stimuli activate MA channels for a rapid influx of ions, such as Ca^2+^, to depolarize the cell membrane, and initiate downstream signaling cascades, including changes in gene expression and protein synthesis. Intracellular Ca^2+^ functions as a second messenger to influence cell responses through modulation of cell proliferation, transcription, protein secretion, and apoptosis (Héraud et al., 2000; Chao et al., 2006; Fodor et al., 2013; Gong et al., 2017; Shen et al., 2021; Zhang et al., 2021). In particular, TRPV4 channels have been shown to influence chondrocyte differentiation, with intracellular Ca^2+^ promoting increased SOX9, collagen II (Col-II), and aggrecan expression (Muramatsu et al., 2007; Wuest et al., 2018).

Since chondrocytes experience an array of mechanical loads, including compression, tension, shear, and hydrostatic and osmotic pressure through extracellular matrix (ECM) and pericellular matrix (PCM), the local composition and stiffness of matrix is altered during OA progression, and, in turn, influences chondrocyte mechanosensitivity and mechanotransduction (Yellowley et al., 1997; Holloway et al., 2004; Buckwalter et al., 2005; Sanchez-Adams et al., 2011; Guilak et al., 2018; Chery et al., 2020). Understanding OA-associated mechanotransduction mechanisms and key mechanotransducers in chondrocytes may provide novel strategies to inhibit or slow the rate of chondrocyte death and ECM degradation that leads to severe OA (Sanchez-Adams et al., 2014). Suspended in cartilage tissue are a few chondrocytes that secrete ECM and regulate tissue homeostasis. Cartilage ECM includes negatively charged proteoglycans, and other molecules like Col-II. The charged nature of proteoglycans attracts water into the matrix, allowing the cartilage to support compressive forces, while Col-II provides tensile strength (Sanchez-Adams et al., 2011; Mardones et al., 2015; Hodgkinson et al., 2021). Immediately surrounding the chondrocyte is the PCM, which can act as a mechanical adaptor to regulate local stress and strain, protecting chondrocytes from large local strains (Korhonen and Herzog, 2008; Wilusz et al., 2013).

In the chondrocyte, Ca^2+^ homeostasis is important in maintaining ECM components and overall health of the cartilage (Wilkins et al., 2003). Disruption of this homeostasis can affect synthesis of ECM molecules and promote catabolism (Guilak et al., 1999; Sánchez and López-Zapata, 2015; Gong et al., 2017). In particular, basic calcium phosphate crystals, found in severe forms of OA, were shown to stimulate chondrocytes by elevating intracellular Ca^2+^. As a result of abnormal Ca^2+^ levels, increased catabolic enzyme production and chondrocyte apoptosis occurred, showing the importance of homeostatic intracellular Ca^2+^ concentrations in maintaining chondrocyte health and cartilage integrity (Nguyen et al., 2012).

It is well established that exercise or physiologic loads promote cartilage anabolism, while traumatic or hyper-physiologic loads trigger cartilage catabolism (Griffin and Guilak, 2005; Guilak, 2011; Ashwell et al., 2013; McCutchen et al., 2017). *In vivo* study of rats demonstrated exercise’s ability to promote DNA repair, ECM synthesis, and suppress ECM degradation enzymes (Blazek et al., 2016). *In vitro* studies reveal that chondrocytes sense applied loads to elicit an appropriate catabolic or anabolic response in strain magnitude-, loading frequency-, and loading duration-dependent manners. For instance, Bleuel et al. showed that chondrocytes under 3–10% strain, 0.17–0.5 Hz, and 2–12 h of stimulation enhances anabolic responses, including increased Col-II and aggrecan expression; and strain, frequency, and duration above 10%, 0.5 Hz, and 12 h, respectively, led to catabolic activity, including upregulation of degradative enzymes like matrix metalloproteinases (MMPs) and downregulation of Col-II and aggrecan expression (Bleuel et al., 2015). Different mechanically activated Ca^2+^ channels in the chondrocyte channelome are the specialized sensors for physiologic or hyper-physiologic loading, initiating specific downstream metabolic responses depending on the magnitude or frequency of a mechanical load. These specific mechano-signaling mechanisms provide potential therapeutic targets for cartilage degeneration. This review summarizes the current understanding of the mechano-signaling mechanisms mediated by TRPV4, Piezo1, and Piezo2 channels in healthy and OA cartilage (Table 1). In addition, we highlight potential therapeutic strategies to halt OA progression.

**TABLE 1:** Selected studies demonstrating mechanosensitive ion channel activity, Ca2+ response to mechanical cues, and biosynthetic activities.

Mechano- sensitive receptor	Channel activity modulation		Model (in vitro/ in vivo)	Ca2+ influx by mechanical cues	Gene expression/ inflammatory response	Ref
	Mechanical	Chemical/Gene				
TRPV4	10% strain, 1 Hz	GSK205 (inhibitor)	Porcine chondrocytes (isolated)	Decreased	Decreased COL2A1, increased ADAMTS5	O'Conor et al. (2014)
		GSK101 (activator)	Porcine chondrocytes (isolated)	Increased	Increased COL2A1, decreased ADAMTS5	O'Conor et al. (2014)
	5 MPa, 0.5 Hz	GSK205	Porcine chondrocytes (isolated)	—	Decrease s-GAG production	Savadipour et al. (2022)
	DMM injury	TRPV4 cKO (Col2a1-CreERT2 x Trpv4lox/lox)	Murine	Decreased	No change in OA progression	O'Conor et al. (2016)
	3% strain, 0.5 Hz or 8% strain, 0.5 Hz	TRPV4 siRNA	Murine chondrocytes (isolated)	Decreased	—	Du et al. (2020)
	10% strain, 0.33 Hz	GSK101, IL-1b	Bovine chondrocytes (isolated)	—	Decreased IL-1b mediated NO and PGE2	Fu et al. (2021)
Piezo1/Piezo 2	—	GsMTx4 (inhibitor)	Porcine chondrocytes (isolated)	Response to 50% strain: decreased	—	Lee et al. (2014)
Piezo1	—	Yoda1 (activator), IL1a	Porcine chondrocytes (isolated)	Increased	Increased PIEZO1 expression, F-actin rarefication	Lee et al. (2021)
	—	Piezo1 siRNA	Human chondrocytes (isolated)	Decreased	Decreased CP-154526- induced cell death	Lawrence et al. (2017)
Piezo2	13% strain, 0.5 Hz or 18% strain, 0.5 Hz	Piezo2 siRNA	Murine chondrocytes (isolated)	Decreased	—	Du et al. (2020)
Piezo2 in nociceptor^a^	DMM injury	Piezo2 cKO (Piezo2-Pdi)	Murine	—	Decrease knee hyperalgesia and NGF- mediated joint nociceptor sensitization	Obeidat et al. (2022)
VGCC	DMM injury	Verapamil (inhibitor)	Murine	—	Increased COL2A1 and ACAN, decreased MMP3	Takamatsu et al. (2014)

a Piezo2 expressed in intra-articular sensory neurons

## Chondrocyte Mechanotransduction Mechanisms

### Cartilage Matrix Homeostasis and OA

Chondrocytes regulate cartilage homeostasis by balancing the synthesis of matrix molecules (Col-II, proteoglycans, aggrecan, etc.) and degrading enzymes (MMPs, ADAMTs, etc.) (Goldring and Marcu, 2009). Physiological loading helps to maintain the integrity of cartilage by decreasing activities of MMPs and suppressing pro-inflammatory factors, but promoting the secretion of more ECM (Wong et al., 1999; Bonassar et al., 2001; Mauck et al., 2003; Goldring and Marcu, 2009; Ng et al., 2009; Leong et al., 2011; Torzilli et al., 2011). Dynamic loading also facilitates transport of molecules throughout the cartilage using convection, which is faster compared to diffusion (Mow et al., 1994; Grodzinsky et al., 2000; Quinn et al., 2001; Evans and Quinn, 2006a; Evans and Quinn, 2006b; Chahine et al., 2009). The transition from healthy to diseased cartilage occurs through an imbalance in the metabolism (catabolic and anabolic reactions) of ECM. Under injurious loading, inflammation promotes enzymatic degradation of ECM proteins through increased MMP activity, resulting in the loss of proteoglycan and other structural matrix components (Sanchez-Adams et al., 2014; Fukui et al., 2015). This matrix degradation alters compressive stiffness and shear resistance of cartilage (Boschetti and Peretti, 2008; Wong et al., 2008; Maier et al., 2019; Mieloch et al., 2019).

In the early phase of OA, these changes are pronounced in the PCM, the extracellular environment immediately surrounding the chondrocyte (Wilusz et al., 2014). In particular, chondrons (chondrocytes and their surrounding PCM) from human OA cartilage experience about 40% reduction in Young’s elastic moduli and 66% more compressive strains than their healthy counterparts (Alexopoulos et al., 2003; Alexopoulos et al., 2005). Aggrecan is synthesized primarily in the PCM and turns over at a faster rate in the PCM than in the surrounding territorial domain ECM (Quinn et al., 1999). During OA related degradation, aggrecan is the first component of the matrix to be degraded (Lark et al., 1995; Han et al., 2011; Chery et al., 2020). Chery et al. performed destabilization of the medial meniscus (DMM) surgery on mouse knees, an injury model of OA progression, and showed that decrease in the PCM compressive modulus occurs about 3-days post-injury, which correlated with a reduction in aggrecan staining seen in the PCM. This decrease in modulus was lower in the PCM than surrounding ECM, suggesting that changes related to OA first occur in the PCM (Guilak et al., 2018; Chery et al., 2020). Compressive modulus in the PCM was further decreased as OA progressed. Blocking of PCM degradation with GM6001, an MMP and aggrecanase inhibitor, lead to an increase in PCM modulus after injury, suggesting PCM integrity at early stages of OA is important to maintaining joint health (Chery et al., 2020). Yet, several experimental therapies targeting MMPs have not been successful in preventing cartilage degradation (Krzeski et al., 2007; Grässel and Muschter, 2020). On the other hand, TRPV4, Piezo1, and Piezo2 channels play a role in Ca^2+^ signaling dependent on substrate stiffness. Specifically, TRPV4 responds to stiffer substrates, while Piezo1/2 to less stiff substrates, making these ion channels potential targets for OA treatment (Du et al., 2021).

### TRPV4-Mediated Mechanotransduction Under Physiologic Loading

**FIGURE 1:**
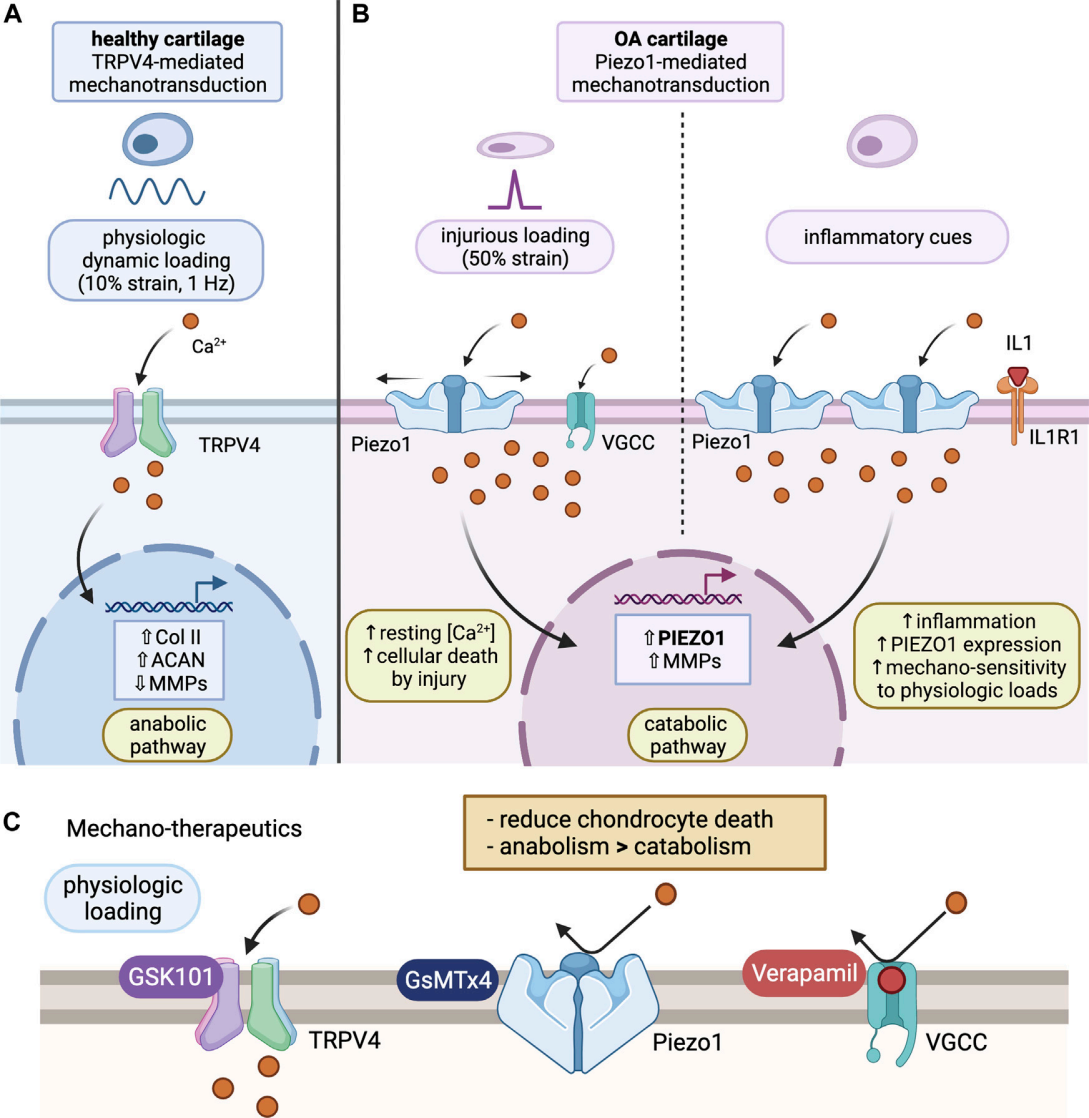
Chondrocyte mechanotransduction and potential mechano-therapeutics. **(A)** TRPV4-mediated Mechanotransduction of Healthy Cartilage: under physiological loading (10% strain), Ca^2+^ ions enter through activated TRPV4 channel and promote an anabolic pathway. This leads to increased collagen II and aggrecan expression, as well as reduced expression of MMPs. Ultimately, this prevents degradation of the cartilage ECM and promotes synthesis of important ECM molecules. **(B)** Piezo1-mediated Mechanotransduction of OA cartilage: under injurious loading (50% strain) or inflammatory activation via IL-1α, Ca^2+^ enters the cell through Piezo1 channels. Activation of Piezo1 channels also triggers voltage gated Ca^2+^ channel opening, resulting in excess Ca^2+^ concentrations in the chondrocyte, activating a catabolic pathway. This will result in enhanced PIEZO1 and MMP expression, increasing mechanosensitivity of chondrocytes to mechanical loading. **(C)** Proposed Mechano-therapeutics: GsMTx4, an inhibitor of Piezo1, prevents Ca^2+^ influx in response to Piezo1 activation under injurious loading, acting to protect the chondrocytes. Verapamil, a VGCC inhibitor, further regulates Ca^2+^ homeostasis by preventing excess Ca^2+^ influx through VGCCs that activate in addition to Piezo1 channels under abnormal loading. GSK101, an agonist of TRPV4, mediates an anabolic phenotype, resulting in reduced expression of degradative enzymes, like MMP, and enhanced expression of cartilage ECM components, like collagen II and aggrecan. Combined, these therapeutics can be used to promote an anabolic pathway, decrease ECM degradation, and prevent progression of the cartilage into an OA phenotype. (Figure created using BioRender.com).

Transient Receptor Potential Vanilloid 4 (TRPV4) is a cation channel that allows influx of Ca^2+^, mediating anabolic responses of chondrocytes triggered by physiological loading (Figure 1A) (Nilius et al., 2003; Hattori et al., 2021); thus, TRPV4 is a potential therapeutic target for OA treatment. TRPV4 is more sensitive to osmotic pressure as a result of increasing charge density by cartilage compression, suggesting that TRPV4 activation is stimulated by osmotic stress transduced from mechanical loading (Sánchez et al., 2014; Lv et al., 2018; Nims et al., 2021). In addition, TRPV4 has been shown to have a delay in Ca^2+^ response after osmotic stimulation (Lv et al., 2018)*. In vitro* experiments involving the use of TRPV4 agonist (GSK101) and antagonist (GSK205) found that TRPV4-mediated Ca^2+^ signaling plays an essential role in the transduction of mechanical stimuli to reinforce and maintain the cartilage matrix and joint health. Physiological loading in this case was defined as 10% strain. TRPV4 activation resulted in an increase in Col-II and sulfated glycosaminoglycans (GAGs) in cartilage. However, chondrocytes with GSK205 in the presence of a mechanical load expressed significantly lower levels of Col-II and higher levels of MMPs (O'Conor et al., 2014; Trompeter et al., 2021a; Savadipour et al., 2022). The effect of TRPV4 activation using GSK101 has been observed to be analogous to that of a mechanical load; chondrocytes treated with GSK101 decrease the synthesis of pro-inflammatory molecules and degradative enzymes (Fu et al., 2021).

TRPV4 has been noted as a possible sensor for excessive stress, resulting in chondrocyte apoptosis (Xu et al., 2019). However, the loading procedure performed in this study was direct stimulation (20% stretch) to the chondrocytes. As TRPV4 channels are usually activated by osmotic stresses, the difference in stimulation mode as well as the hyper-physiological strain on the chondrocytes may have caused cell death, a different effect than the usual anabolic pathway that TRPV4 mediates under physiologic loads.

*In vivo* experiments have also highlighted the essential role of TRPV4 channels for cartilage health and disease. Mice with cartilage-specific TRPV4-deletion in adulthood exhibit reduced severity of aging-associated OA compared to control mice; however, analysis following DMM injury show similar levels of cartilage degradation and OA severity between control and TRPV4-deficient mice (O'Conor et al., 2016). This suggests that age-related and post-traumatic osteoarthritis (PT-OA) are mediated through distinct pathways. As TRPV4 inhibition did not prevent the progression of OA after injury, it is not a suggested therapeutic strategy for PT-OA treatment (O'Conor et al., 2016). The reduced aging-OA phenotype in cartilage of TRPV4-deleted mice may be due to imbalanced matrix metabolism, or redundancy in the mechanotransduction pathways that may compensate for TRPV4-deletion (Servin-Vences et al., 2017). In short, these collective data demonstrate the essential roles of TRPV4 in cartilage maintenance and anabolism (O'Conor et al., 2014).

### Piezo1/Piezo2-Mediated Mechanotransduction Under Injurious Loading

Piezo1 and Piezo2 channels are mammalian-expressing mechanosensitive cation channels discovered in 2010 that allow passage of Ca^2+^ into chondrocytes (Coste et al., 2010; Coste et al., 2012). Both Piezo1 and 2 channels are directly and rapidly activated by mechanical cues (τ_ac_Piezo1_ < 5 msec) with rapid subsequent inactivation time (τ_inac_Piezo1_∼ 16 msec, τ_inac_Piezo2_ ∼ 7 msec) (Coste et al., 2010). Yet, these channels have distinct gene expression patterns and are associated with different types of human diseases. Piezo1 is robustly expressed in mechanically stimulated tissues, including lung, colon, bladder, kidney, blood vessels, and in cells, including red blood cells, cardiac fibroblasts, and smooth muscle cells (Xu et al., 2020). Activated by mechanical forces at the cell membrane, Piezo1 channels mediate responses in the cell, such as adjusting cell volume or remodeling host tissue, through activation of intracellular signaling pathways. Mutations in the Piezo1 channel are associated with lymphatic dysplasia and hemolytic anemia (Syeda et al., 2015; Xu et al., 2020). In contrast, Piezo2 channels are highly expressed in sensory systems, including proprioceptive mechanosensors and Merkel cells, controlling limb movement and touch sensation (Xu et al., 2020; Fang et al., 2021). Mutations in Piezo2 lead to muscular atrophy, distal arthrogryposis, and scoliosis arthrogryposis (Anderson et al., 2017; Assaraf et al., 2020).

Articular chondrocytes express both Piezo1 and Piezo2 channels (Piezo1/2) robustly, and both channels are key mechanotransducers sensing injurious level (high-strain) mechanical loads (Lee et al., 2014; Du et al., 2020). Compression with a strain of ∼50% by atomic force microscopy (AFM) probes on isolated chondrocytes leads to a significant and prolonged intracellular Ca^2+^ influx with τ_inac_chondrocyte_ ∼ 16 s (not msec). These robust Ca^2+^ transients were diminished in chondrocytes with either Piezo1-knockdown or Piezo2-knockdown, as well as with GsMTx4 (an inhibitor of both Piezo1 and Piezo2) or verapamil [an inhibitor of L-type voltage-gated Ca^2+^ channels (VGCC)] treatment. These data suggest the synergistic action of Piezo1 and Piezo2 in transducing mechanical signals, and the role of VGCC in amplifying intracellular Ca^2+^ after Piezo1/2 activation (Figure 1B). The synergistic activation of Piezo1 and Piezo2 channels were further seen in heterologous cells with co-transfection of Piezo1 and Piezo2 under AFM-based compression or electrophysiology-based membrane stretch, but not in model cells with only-Piezo1 or only-Piezo2 transfections (Lee et al., 2014). The chondroprotective effect of Piezo1/2 inhibition using GsMTx4 was shown in a cartilage explant injury model, where porcine osteochondral explants were injured with a biopsy punch, resulting in chondrocyte damage at the area of injury. GsMTx4 pre-incubation of the explants was shown to decrease the “zone of death,” or damaged area, demonstrating GsMTx4’s effect of protecting chondrocytes from mechanical trauma via Piezo1/2 inhibition (Lee et al., 2014; Lawrence et al., 2017). GsMTx4 will be explored further as a potential therapeutic in a later section.

### Role of Piezo1 in Inflammatory Signaling of Chondrocytes

Osteoarthritic joints and acutely injured joints exhibit significantly increased levels of interleukin-1 (IL-1) cytokines with enhanced inflammatory signaling in chondrocytes. Chondrocytes express functional IL-1 receptor (IL1R) and respond to both isoforms of IL-1α and IL-1β (Martel-Pelletier et al., 1992; McNulty et al., 2013). IL-1α-treatment increases Piezo1 preferentially, but not Piezo2 or TRPV4 channels, in primary articular chondrocytes. Chondrocytes in porcine and human OA cartilage also express 2-fold Piezo1 channels compared to healthy cartilage (Lee et al., 2021). Piezo1 augmentation further increases hyper-mechanosensitivity of chondrocytes *in vitro* (Figure 1B). AFM-based assay data reveal the increased Ca^2+^ influx from cyclic physiologic loading in IL-1α-treated or Yoda1 (a Piezo1-specific agonist) chondrocytes compared to controls, which in turn was diminished by co-treatment with Piezo1-siRNA or GsMTx4. These data suggest Piezo1’s role in the inflammatory response, disrupting Ca^2+^ homeostasis and increasing mechano-sensitivity of chondrocytes to mechanical loads.

Inflammation also affects the cytoskeleton, particularly filamentous actin (F-actin), as force transduction through F-actin is important in chondrocyte mechanotransduction (Wang et al., 1993; Haudenschild et al., 2008; Trompeter et al., 2021b; Dieterle et al., 2021). With exposure to IL-1α, F-actin of primary chondrocytes was reduced—an effect that was also seen in human OA cartilage samples. However, F-actin was restored with Piezo1 inhibition via GsMTx4 or Piezo1-targeting siRNA. Exposure to IL-1α also resulted in a decrease in cellular Young’s modulus, leading to increased cellular deformation with the same magnitude of mechanical loading compared to control samples. Inhibition of Piezo1 returned the cellular modulus and cell deformation to control levels. This shows that influx of Ca^2+^ through Piezo1 can affect cytoskeletal components including F-actin, resulting in a decrease in mechanical stiffness of the chondrocyte, increasing the likelihood of tissue degeneration. IL-1α-treatment also augmented Piezo1 via p38-MAPK signaling pathways and ATF2/CREBP1/HNF4 transcription factors (TFs). Testing of MAP-kinases downstream of IL1R showed that inhibition of p38-MAP kinase led to a decrease in Piezo1 mRNA expression with IL-1α exposure. Screening for TFs showed that inhibition of ATF2/CREBP1 and HNF4 attenuated Piezo1 mRNA expression in response to IL-1α.

Altogether, inflammatory cytokine IL-1α activates IL1R, where the signal is transduced by p38-MAPK, resulting in the activation of Piezo1 expression through TFs, ATF2/CREBP1 and HNF4. The increased expression of Piezo1 can result in increased Ca^2+^ influx, resulting in the loosening of the F-actin network (Lee et al., 2021). This can decrease cellular stiffness, and in turn decrease tissue stiffness, increasing the chance of developing an OA phenotype.

### OA-Mediated Pain and the Role of Piezo2 in Joint Nociception

Piezo2 channels expressed in intra-articular sensory neurons have been studied in the context of nociception, mediating inflammation and nerve injury-induced sensitized mechanical pain or mechanical allodynia. Piezo2 expression is high in low threshold mechanoreceptors, likely contributing to their sensitivity to mechanically activated pain. Knockout of Piezo2 in mice impaired nociceptor firing, resulting in disrupted responses to noxious mechanical stimuli (Murthy et al., 2018). In a study by Szczot et al., Piezo2 mediated inflammation-induced pain in tactile allodynia. With Piezo2 knockout, mice failed to develop sensitization and pain in response to touch after skin inflammation, suggesting a possible role of Piezo2 in mediating pain sensation under inflammation (Szczot et al., 2018).

There has been ongoing investigation of the role of Piezo2 in OA mediated pain, however, the mechanism of this pain transduction pathway is not yet completely understood. Miller et al. studied pain reactions in mice with homozygous or heterozygous Piezo2 deletion in a DMM surgery-induced mouse OA model. In wild-type mice with intact Piezo2, knee hyperalgesia and mechanical allodynia of the ipsilateral hind paw developed 4-weeks post-surgery. Less mechanical allodynia was seen with heterozygous deletion of Piezo2 4-weeks after DMM, however, knee hyperalgesia did not change compared to the mice with intact Piezo2. In mice with Piezo2 homozygous deletion, less knee hyperalgesia and mechanical allodynia was seen in the hind paw at 4-weeks post-DMM surgery (Miller et al., 2019). In further study, it was shown that conditional knockout of Piezo2 in mice lead to attenuated nerve growth factor (NGF)-mediated knee swelling and mechanical pain (Obeidat et al., 2022). These data suggest an essential role of Piezo2 in mediating OA-associated joint nociceptor sensitization.

## Current and Potential Mechano-Therapeutic Strategies

### Current Therapeutic Strategies

The goal of OA therapeutics is to prevent progressive cartilage degeneration and joint dysfunction. OA therapeutics are urgently needed especially for younger patients who have a high risk for PT-OA a decade after joint injury (Anderson et al., 2011; Schenker et al., 2014; Krishnan and Grodzinsky, 2018; Eskelinen et al., 2020). Exercise and physical therapies are currently suggested after surgery to promote anabolism, presumably by targeting the TRPV4 ion channels. In addition to exercise, patients may receive intra-articular injections of hyaluronic acid (HA) or corticosteroids, to increase cartilage lubrication or decrease local inflammation, respectively. HA is a GAGs found in the cartilage and synovial fluid, which provides the joint with lubrication and shock absorbance (Fusco et al., 2021). With OA progression, HA in the synovial fluid usually depolymerizes from high to low molecular weight, resulting in a decline in mechanical and viscoelastic properties of the joint. Exogenous injection of HA can promote synthesis of extracellular matrix proteins, proteoglycans, and/or GAGs, and have anti-inflammatory effects (Bowman et al., 2018). Usually used for short-term pain-relieving treatment, HA injection has shown to provide some pain relief, however, injections are expensive (Trigkilidas and Anand, 2013; Liu et al., 2018). Some patients also receive intra-articular injections of corticosteroids, which have immunosuppressive and anti-inflammatory effects on the joint, blocking synthesis of pro-inflammatory molecules (IL-1) and catabolic proteins (MMPs). Patients with joint inflammation caused by OA benefit more with corticosteroid injection, compared with HA. However, corticosteroid treatment provides only temporary, short term pain relief (Ayhan et al., 2014; Fusco et al., 2021; Primorac et al., 2021).

The above therapies are limited in that they only control the symptoms of OA after disease onset and progression, and they are used as conservative therapies before the need for surgical intervention. Disease-modifying OA drugs (DMOAD) are currently being investigated with the goal to halt cartilage degradation, promote matrix regeneration, and reduce OA-mediated pain. This includes therapies aiming to inhibit MMPs, like MMP-13 and aggrecanases, to prevent the degradation of cartilage matrix that occurs in OA (Kurz et al., 2005; Chubinskaya et al., 2015). A clinical trial was conducted for MMP inhibitor PG-116800 to test its ability to delay cartilage destruction. PG-116800 has high affinity for MMP-2, -3, -8, -9, -13, and -14, and low affinity for MMP-1 and MMP-7. The trial was terminated due to a musculoskeletal toxicity side effect. There was also no significant difference in radiographic knee joint space between treatment and placebo groups, suggesting the therapy was ineffective in preventing degradation of cartilage. The major adverse effect seen was arthralgia. The investigators hypothesized that the musculoskeletal symptoms may have been due to MMP inhibitors ability to inhibit sheddase activity, which normally converts cytokines into inactive forms (Krzeski et al., 2007). Inhibition of this activity would then result in paradoxical inflammation. Another hypothesis made was that the toxicity was due to MMP-1 inhibition. Efforts to develop a selective compound to target inactivation of only MMP-13 are in progress, but results are forthcoming (Baragi et al., 2009; Vandenbroucke and Libert, 2014; Li et al., 2017).

Other therapies targeting inflammatory cytokines active in OA, like IL-1, IL-6, and TNF-α, have been studied as well (Jacques et al., 2006; Chubinskaya et al., 2015). Most of these therapeutics were originally developed for treatment of rheumatoid arthritis (RA) and adapted to treat OA. However, these treatments were ultimately ineffective in preventing pain or cartilage degradation. An example is anakinra, an IL-1 receptor antagonist. Investigators attributed this lack of effectiveness due to the mode of treatment administration, a single intra-articular injection to the knee (Chevalier et al., 2009). Further studies are anticipated to investigate more long-lasting, potent IL-1 receptor antagonists (Jotanovic et al., 2012).

Senescent cells have also been targeted since these cells are accumulated in areas of cartilage degeneration in OA. Senescence has been shown to promote oxidative stress and inflammation in diseased cartilage (Vinatier et al., 2018). A senolytic molecule, UBX0101, was developed to remove these cells by inhibiting MDM2/p53 interactions, reducing the release of inflammatory factors and associated pain (Loeser, 2011; Collins et al., 2018; Hsu et al., 2019; Coryell et al., 2021). However, this trial of UBX0101 intra-articular injection was discontinued as it failed to meet week-12 primary endpoints, with no significant difference between treatment and placebo groups (Hsu et al., 2019; Grässel and Muschter, 2020).

### Potential Therapy: GsMTx4 Peptide Therapy Targeting Piezo1

GsMTx4, a 34 amino acid peptide derived from tarantula venom, inhibits Piezo1 and Piezo2 channels (Bowman et al., 2007; Gottlieb et al., 2007; Copp et al., 2016; Alcaino et al., 2017). GsMTx4 anchors to the outer membrane surface by lysine residue at low tension. When the membrane is under tension, GsMTx4 is able to physically sink deeper into the membrane, leading to partial relaxation of the outer monolayer of the membrane. This disrupts the distribution of tension near mechanosensitive channels including Piezo1, causing a less efficient transfer of force from the bilayer to the channel without physical block of ion pore regions. The change in membrane tension creates a 30 mmHg rightward shift in the pressure-gating curve, making it harder for Piezo1 to open under mechanical stimulation (Bae et al., 2011; Gnanasambandam et al., 2017). GsMTx4 has been shown to be ineffective in inhibiting TRPV4 channels, demonstrated in juxtaglomerular cells, bladder urothelium, and endothelial cells (Seghers et al., 2016; Ihara et al., 2018; Swain and Liddle, 2021). In chondrocytes, GSK205 (a TRPV4 inhibitor) failed to inhibit Ca^2+^ influx under hyperphysiological loading, while GsMTx4 treatment did, further confirming GsMTx4’s ability to selectively inhibit Piezo channels. A possible mechanism as to why GsMTx4 is specific to Piezo may be due to the unique structure of these channels. Piezo channels have three curved, blade-like structures that widen from the base of the protein to the mechanosensing portion on the outer layer of the plasma membrane (Fang et al., 2021). This may allow GsMTx4 to embed closer to the Piezo channel and influence membrane tension more locally, effecting Piezo channel activation specifically.

The use of GsMTx4 has been studied in the treatment of Duchenne muscular dystrophy (DMD). DMD is caused by genetic mutation resulting in a loss of dystrophin, which is linked to increased permeability of the sarcolemma to extracellular Ca^2+^. This leads to a decline in muscle mass due to increased Ca^2+^-dependent proteolysis and necrosis of muscle fibers. GsMTx4-D, an enantiomer of GsMTx4, was shown to decrease loss in muscle mass and improve the muscle’s functional capacity due to inhibiting mechanically stimulated channels like Piezo1 (Suchyna, 2017). Ward et al. studied the pharmacokinetics of GsMTx4 in mice. Through 50 mg/kg dose subcutaneous injection, GsMTx4 accumulation of 0.1–5 μM in skeletal muscle and heart was achieved within 24 h, a range shown to effectively limit MA channel activity. GsMTx4 also demonstrated long half-life in tissues, but rapid depletion in the blood, suggesting higher affinity of GsMTx4 for tissues than serum proteins. D-amino acid peptides are less prone to enzymatic degradation, which may contribute to GsMTx4-D’s long half-life. No apparent adverse effects or signs of toxicity were shown during the 6-weeks study in mice, although further study of long-term effects, particularly on growth and development, would be needed (Ward et al., 2018).

A cardio-protective effect was also seen with use of GsMTx4 in the context of cardiac ischemic reperfusion injury, which is often associated with an elevation of Ca^2+^ influx. Wang et al. showed that mice with intravenous injection of GsMTx4-D during an ischemic event or with subcutaneous injection prior to ischemic challenge show reduced infarct size, less arrhythmic activity, and increased cardiac output post ischemia. GsMTx4 treatment also improved heart contraction by restoring normal Ca^2+^ release and blocked apoptotic signaling to improve cardiomyocyte survival. Slowing of cation influx through ion channels with GsMTx4 during ischemia and reperfusion prevented cell swelling that occurs with cation overload. GsMTx4 was mostly active at pathological conditions, as there was little effect of the treatment on normally functioning controls (Wang et al., 2016).

In the context of OA, GsMTx4-treated cartilage demonstrates a chondroprotective effect in hyper-physiological loading by inhibiting Piezo1 and Piezo2 channels. Osteochondral cartilage explants with pre-incubation of GsMTx4 showed significantly decreased chondrocyte damage and death after biopsy punch injury (Lee et al., 2014). GsMTx4 was also shown to prevent inflammation-induced rarefication or loosening of F-actin, an important cytoskeleton component in chondrocyte mechanotransduction. Inhibition of the Piezo1 channel via GsMTx4 preserved the cellular modulus in the presence of IL-1α as well (Lee et al., 2021). To date, the effect of GsMTx4 in the context of articular cartilage injury has been studied in *in vitro* and *ex vivo* models. Moving forward, further study would be needed to see whether the chondroprotective effect translates to *in vivo* animal models and potential clinical use. Along with this, appropriate dosing for intra-articular injection of GsMTx4 would need to be determined, as well as any potential toxicities related to long term use of GsMTx4. Based on its application to treatment of other disease, GsMTx4 seems to be nontoxic and effective in treating pathologies related to Piezo1 channel dysfunction.

### Potential Therapy: Verapamil Targeting VGCC

As an FDA-approved drug, verapamil has been used in the treatment of various cardiac conditions including angina, arrhythmias, and hypertension, with no major adverse effects observed (Brogden and Benfield, 1996; De Simone et al., 2003). A commonly prescribed L-type voltage-gated calcium channel (VGCC) blocker, verapamil has also been studied as a therapeutic to attenuate Wnt/β-catenin signaling in OA (Matta et al., 2015; Vaiciuleviciute et al., 2021). The activation of β-catenin can induce hypertrophic differentiation of chondrocytes and upregulate ECM catabolic enzymes, leading to development of an OA-phenotype (De Santis et al., 2018; Wang et al., 2019; Lories and Monteagudo, 2020). Verapamil is able to suppress Wnt/β-catenin signaling by enhancing FRZB gene expression, an antagonist of Wnt signaling, which leads to suppressed ECM degradation (lower MMP activity), enhanced gene expression of aggrecan and Col-II, and decreased hypertrophic differentiation of chondrocytes (lower type X collagen expression). In a study by Takamatsu et al., 50 μM of verapamil was delivered to rats intra-articularly after DMM, preventing progression of OA without apparent adverse effects, although long term use in clinical practice needs further investigation (Takamatsu et al., 2014).

In their investigation of chondrocytes, Lee et al. suggest that Piezo1 activation may lead to activation of VGCCs, amplifying intracellular Ca^2+^ signaling in response to injurious loading. Verapamil was shown to decrease the Ca^2+^ transients in response to injurious compression, suggesting that VGCCs may be activated in addition to Piezo1 with hyper-physiological loading, as opposed to Ca^2+^ movement via TRPV4 in response to hypo-osmotic stress (Lee et al., 2014; Nims et al., 2021). This may indicate a correlation between Piezo1, VGCCs and Wnt signaling which are all activate during injurious loading. Further study is needed to confirm Piezo1’s direct effect on Wnt signaling in chondrocytes.

### Future Direction

Targeting OA-associated chondrocyte mechanotransduction shows promise as future therapeutics for OA. Based on current knowledge, OA therapeutic strategies would be to promote TRPV4-mediated cartilage anabolism and to inhibit Piezo1-mediated chondrocyte death and inflammatory feed-forward responses. These strategies may be achieved by administrations of GSK101, GsMTx4, and verapamil (Figure 1C). Intra-articular injections are suggested to specifically target tissues in synovial OA joints, reducing systemic side effects to other organ systems, in addition to increasing the drug’s bioavailability. The use of mechanoresponsive biomaterials can further control the delivery of drugs (Geiger et al., 2018). For example, nanoparticles containing these drugs may release its contents into the joint space over time, generating a sustained release. Release of a drug can also be controlled based on compressive, tensile, or shear forces applied to a hydrogel containing the drug. Specifically, in this application, a hydrogel may be tuned to release GsMTx4 under hyper-physiological loads (ex. >300 nM compression), thus, releasing the drug only as needed. This technology may increase the longevity of a single treatment and reduce overall treatment costs over time (Hodgkinson et al., 2021).

## Conclusion

TRPV4-, Piezo1-, and Piezo2-mediated mechanotransduction mechanisms of chondrocytes play essential roles in cartilage regeneration and degeneration. Our understanding of the specific mechanosignalling pathways and downstream signals of these mechanosensitive Ca^2+^ channels yield potential safe and efficient OA treatments. Potential mechano-therapies include activating TRPV4-mediated mechanotransduction and inhibiting Piezo1-mediated mechanotransduction to promote cartilage anabolism and prevent cartilage catabolism or degradation. The continued advances in chondrocyte mechanobiology will lead to successful DMOADs with long-term safety to restore cartilage integrity for OA patients.
